# Thyroid Storm in a Patient on Chronic Amiodarone Treatment

**DOI:** 10.7759/cureus.24164

**Published:** 2022-04-15

**Authors:** Muhammad Ali Raza, Ashish Jain, Maha Mumtaz, Talha Mehmood

**Affiliations:** 1 Internal Medicine, Conemaugh Memorial Medical Center, Johnstown, USA

**Keywords:** amiodarone-induced hyperthyroidism, ait type 2, ait, thyroid storm from drugs, amiodarone thyroid storm

## Abstract

Amiodarone is a class III antiarrhythmic drug with a structure comparable to thyroid hormone and 37% iodine content by weight. In addition to direct drug cytotoxicity on thyroid cells, amiodarone deiodination in the body releases an excessive amount of iodine, which can impair thyroid function and cause hypothyroidism or thyrotoxicosis, including thyroid storm in susceptible individuals. In this report, we discuss the case of a 52-year-old woman who experienced a thyroid storm after being treated with amiodarone, eventually leading to her death.

## Introduction

A thyroid storm is a metabolic outburst with significant mortality caused by the thyroid gland’s rapid and uncontrolled release of thyroxines. The mortality rate of thyrotoxicosis combined with thyroid storm is 12 times higher than that of thyrotoxicosis alone [1]. Amiodarone has a long history of being linked to thyroid diseases (hypothyroidism and hyperthyroidism), which is due to a high concentration of iodine in the drug’s formulation having a damaging effect on thyroid follicles. Supportive care with corticosteroids, beta-blockers, and thioamides is the mainstay of treatment [2].

## Case presentation

A 52-year-old bedridden female with a history of Friedreich’s ataxia, neurogenic bladder on an indwelling Foley catheter, cardiomyopathy, intellectual disability, and atrial fibrillation on amiodarone 200 mg/day for the previous four years presented to the emergency department (ED) with low-grade fever, palpitations, swelling of her face and extremities, and altered sensorium. Because of her memory problems and impaired mentation, further review of systems was limited. Initial vitals revealed a temperature of 99.8°F, tachycardia with a heart rate of 112 beats per minute, respiratory rate of 16 breaths per minute, blood pressure of 86/49 mmHg, and oxygen saturation of 97% on ambient air. The patient appeared ill, with facial and extremity edema and severe muscle atrophy. She was awake, but not oriented to time and place. A cardiac examination revealed normal S1-S2; however, the patient had sinus tachycardia. There were scattered rhonchi bilaterally on lung examination, but no wheezing or use of accessory muscles. She also had a small ulcer over her sacrum with cellulitic changes all around it. Reduced muscle mass and strength were noticed during a nervous system examination.

**Figure 1:**
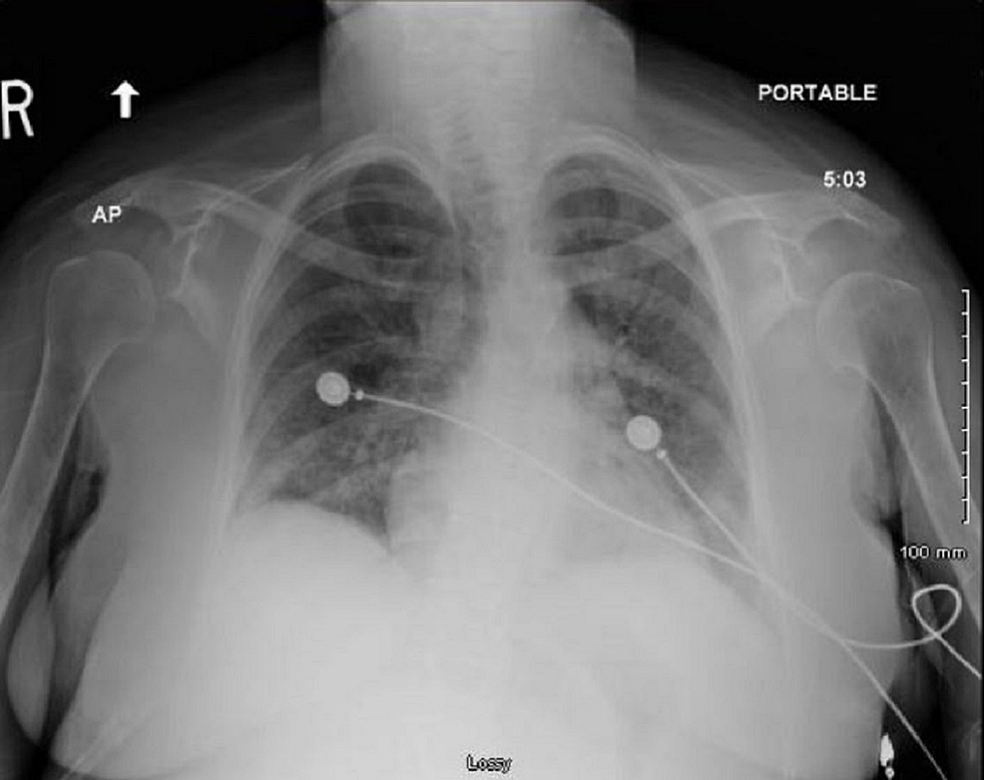
Chest X-ray of the patient showing diffuse and bilateral interstitial edema.

Her initial blood work revealed leukocytosis, as well as increased troponins and brain natriuretic peptide (BNP). Given her altered mentation, the patient was intubated in the ED and started on vasopressors before being transferred to the intensive care unit (ICU). The patient developed atrial fibrillation with a rapid ventricular rate in the 150s and 160s on her first day in the ICU, requiring synchronized cardioversion because of hemodynamic instability. The hepatic function panel revealed transaminitis with elevated aspartate aminotransferase 736 U/L (reference: 5-34 U/L) and alanine transaminase 117 U/L (reference <54U/L). Her chest X-ray showed diffuse and bilateral interstitial opacities likely pulmonary edema or pneumonitis (Figure 1).

The patient was hyperthyroid biochemically with a low thyroid-stimulating hormone of 0.11 uIU/mL (reference: 0.35-5.5 uIU/mL) and high free T4 of 2.44 ng/dL (reference: 0.9-2.3 ng/dL). Because of the strong suspicion, the Burch criteria score for thyroid storm was calculated, which was 60, and was highly suggestive of thyroid storm associated with type II amiodarone-induced hyperthyroidism (AIT), given the absence of past thyroid disease and long-term amiodarone treatment (Table 1).

**Table 1:** Burch Criteria Score in our patient.

Burch-Wartofsky point scale factor	Patient’s status	Patient’s score
Thermoregulatory dysfunction (temperature)	99.8°F	5
Heart rate (beats per minute)	112	10
Atrial fibrillation	Present	10
Congestive heart failure	Mild (pedal edema)	5
Central nervous system effects	Moderate (extreme lethargy)	20
Gastrointestinal-hepatic dysfunction	Absent	0
Precipitating event	Present (amiodarone)	10
Total score		60

The patient was started on intravenous (IV) hydrocortisone 300 mg, followed by 100 mg every eight hours, in addition to supportive care with IV fluids. The patient was not given propylthiouracil because of her hepatic impairment. The endocrinologist was consulted who advised a single dose of methimazole 20 mg. The patient developed anuric acute kidney injury as a result of the profound hypotension and was immediately started on continuous renal replacement therapy as per nephrology recommendations. She also became highly acidotic, requiring multiple ampules of bicarbonate and pressors such as epinephrine, norepinephrine, phenylephrine, and vasopressin. The patient’s family was informed of her critical condition and the code status was changed to do-not-resuscitate, followed by comfort measures as per her family’s wishes.

## Discussion

A thyroid storm is described as a hypermetabolic state caused by unregulated thyroid hormone release. In 1993, the Burch-Wartofsky point scale was introduced for the risk prediction of thyroid storm, factors included in the scale were temperature, central nervous system dysfunction, tachycardia, atrial fibrillation, degree of heart failure, gastrointestinal dysfunction, and presence of precipitating factor. A total score of more than 45 is highly suggestive of a thyroid storm [3]. Amiodarone has been historically associated with thyroid disorders (hypothyroidism and hyperthyroidism) which is due to a high concentration of iodine in the drug’s formulation and its toxic effect on thyroid follicles. AIT has been classified into two types based on its pathology, management, and outcomes. Type 1 AIT is characterized by excessive thyroid hormone production, while type 2 AIT is caused by an excess release of the preformed hormone as a result of thyroid follicle destruction [4]. A case series involving 13 patients found that thyroid storm was the first clinical presentation of thyrotoxicosis and 46.4% of patients with an overall mortality rate of 25%. The study also found predominant cardiac involvement in >60% of the patients presenting with atrial fibrillation and severe tachycardia (heart rate >140 beats per minute). Although central nervous system involvement was less common (28.6%), it was statistically linked to increased mortality [5].

Thyroid storm is treated with supportive measures and symptomatic care, such as IV fluids and oxygen, as well as specific treatments to treat hyperthyroidism and prevent additional thyroid gland damage. Patients with thyroid storm should be transferred to the ICU, where they will be closely monitored and provided ventilatory support. For symptomatic management of any suspected thyroid storm, a beta-blocker (esmolol) might be administered after initial supportive measures. Hydrocortisone 100 mg IV every eight hours or dexamethasone 2 mg every six hours are the mainstay treatments [6]. Along with vascular and hormonal support, this may be effective in preventing adrenal insufficiency and reducing vasomotor symptoms. Glucocorticoids reduce peripheral T4 to T3 conversion, therefore, reducing the occurrence of hypermetabolic symptoms [6]. Aside from that, long-term use of thioamides and treating the precipitating or underlying stimuli can lower mortality rates [7]. The efficacy of plasmapheresis in patients with thyroid storms and its influence on reducing free T3 and free T4 levels was reported by Simsir et al. [8]. Zhu et al. discussed the possible role of plasmapheresis in the treatment of thyroid storms caused by type II AIT [9].

## Conclusions

A thyroid storm is a rare endocrinological emergency with high mortality. Type II AIT is rarely associated with thyroid storm and can pose management challenges, especially in patients with multiple comorbidities.
